# Electrochemical Performance of Corn Waste Derived Carbon Electrodes Based on the Intrinsic Biomass Properties

**DOI:** 10.3390/ma16145022

**Published:** 2023-07-15

**Authors:** Kunhan Xie, Wen Zhang, Kai Ren, Enze Zhu, Jianyi Lu, Jingyang Chen, Penggang Yin, Liu Yang, Xiaohui Guan, Guangsheng Wang

**Affiliations:** 1 Jilin Provincial Science and Technology Innovation Center of Clean Conversion and High-Valued Utilization of Biomass, School of Chemical Engineering, Northeast Electric Power University, Jilin 132012, China; 2 School of Chemistry, Beihang University, Beijing 100191, Chinawanggsh@buaa.edu.cn (G.W.)

**Keywords:** corn cob and corn silk derived carbon materials, biomass properties, composition and structure regulation, electrochemical performance optimization, theoretical and experimental investigations

## Abstract

The exploration of cost-effective and sustainable biomass-derived carbon materials as electrodes for energy conversion and storage has gained extensive attention in recent research studies. However, the selection of the biomass and the electrochemical performance regulation of the derived biochar, as well as their interrelationship still remain challenging for practical application. Herein, corn wastes with high carbon content (>40%), corn cob and corn silk, were selected as precursors for the preparation of high value-added and high yield carbon materials via a modified synthetic process. Uniquely, this work put emphasis on the theoretical and experimental investigations of how the biomass properties influence the composition and nanostructure regulation, the electrolyte ion adsorption free energy, and the electrical conductivity of the derived carbon materials as well as their electrochemical performance optimization. Owing to the favorable specific surface area, the hierarchical porous structure, and the diverse elemental distribution, corn cob and corn silk derived carbon materials (CBC and SBC) present great potential as promising electrodes for alkaline aqueous zinc batteries and supercapacitors. The assembled CBC//Zn and SBC//Zn zinc batteries deliver high energy densities of 63.0 Wh kg^−1^ and 39.1 Wh kg^−1^ at a power density of 575 W kg^−1^, with excellent cycling performance of 91.1% and 84.3% capacitance retention after 10,000 cycles. As for the assembled symmetric supercapacitors, high energy densities of 14.9 Wh kg^−1^ and 13.6 Wh kg^−1^, and superior long-term cycling stability of 99.3% and 96.6% capacitance retention after 20,000 cycles could be achieved. This study highlights the advantages of utilizing corn cob and corn silk as carbon sources on the designed synthesis of carbon electrodes, and presents a meaningful perspective in the investigation of biomass-derived carbon materials and their potential applications in rechargeable devices.

## 1. Introduction

The development and utilization of renewable energy sources have gained increasing attention due to the rapid depletion of fossil fuels and the associated severe environmental pollution problems [1,2]. As a result, the optimization of high-performance energy storage devices has become a top priority to realize the application of clean energy in particular fields [3]. Among numerous energy conversion and storage devices, zinc batteries and supercapacitors have attracted considerable attention in addressing energy demands and environmental concerns [4,5,6,7,8], while the development of high-performance electrode materials has been shown to be a feasible and effective method to control and regulate the charge storage capability of the rechargeable devices.

Various carbon materials, such as biochar [9,10], carbon nanotube [11,12], graphene [13,14], and activated carbon [15,16,17], have been explored as electrode materials. Generally, they have characteristics of great rate performance and cycling stability [18,19]. However, their electrochemical performance is still quite different, resulting from the differences among their nanostructures and compositions. Considering the charge storage mechanism, the accessible specific surface area, suitable pore structure, and abundant active sites become the chief characteristics for an ideal carbon electrode [20,21,22]. Biochar is derived from the carbonization or graphitization of biomass, which is widely available and environmentally friendly [23,24]. Recently, the research studies on converting biomass into various functional carbon materials for energy storage have received great attention [25,26]. Ma et al. prepared porous biochar by a typical pyrolysis method combined with chemical activation using tea saponin as the precursor. With the rational modulation of the KOH dosage, the as-prepared carbon material exhibited desirable nanostructure and interface properties, which led to a high specific capacitance of 278 F g^−1^ and excellent cycling stability [27]. After proper treatment, the obtained biochar with rich pore structure and heteroatom doping could provide sufficient space and active sites for the free migration and adsorption of electrolyte ions, and ultimately effectively improve the charge storage capacity of materials [28,29,30]. In addition, it is crucial to select suitable biomass for the preparation of biochar-based electrode materials [31], and as such more and more agricultural wastes have been employed as precursors [32,33]. For example, Purkait T et al. proposed a scalable mechanical exfoliation method to prepare few-layer graphene-like nanosheets from peanut shell, the obtained electrode exhibited a high specific capacitance of 186 F g^−1^ in H_2_SO_4_ electrolyte (1 mol L^−1^) [34]. Ma et al. employed sorghum stalk as raw material to produce porous carbon via a universal carbonization method. The derived carbon electrode showed a high specific capacitance of 216.5 F g^−1^ at 0.5 A g^−1^ and excellent cyclic stability with 92% capacitance retention after 5000 cycles. Specifically, the doping of N and O atoms greatly increased the electrochemical active sites of the electrode material, which were beneficial for the optimization of its holistic electrochemical performance [35].

Among the numerous crops, corn, as one of the largest grain species in the world, produces numerous biomass wastes every year. Although they are biodegradable, the superior management of the wastes into high-valued products is desirable [36]. In this research, the corn wastes, corn cob and corn silk were selected as the precursors to synthesize high-performance porous carbon materials, which were labeled as CBC and SBC, respectively. With a mild and feasible chemical activation method, the yield of the products could be guaranteed, and the as-prepared CBC and SBC deliver high specific capacitances of 283.8 F g^−1^ and 266.0 F g^−1^ at the current density of 1 A g^−1^, and superior cycle stability of 85.3% and 86.1% after 10,000 cycles, respectively. In addition, the high-performance CBC and SBC were used as the positive electrodes in zinc batteries and supercapacitors, which possess obviously higher energy and power density, as well as cycling stability than most reported biochar electrode based rechargeable devices. Furthermore, the influences of the biomass properties on the composition and nanostructure regulation of the derived carbon materials and their electrochemical performance optimization were also investigated. This study fully proved the advantages of utilizing corn cob and corn silk as precursors to controllably synthesize high-valued carbon-based electrodes, which are expected to become commercial on a large scale. The prepared biochar-based electrodes were used creatively in the zinc batteries and proved to have great potential.

## 2. Experimental Section

### 2.1. Materials

All the chemicals were of analytical grade and used as received without any further purification. The water used in this research was ultrapure water.

### 2.2. Synthesis of the CBC and SBC Materials

Corn cob and corn silk derived carbon materials with hierarchical porous structure were prepared via a pyrolysis process with KOH as the activating agent. The crushed corn cob and corn silk were first screened through 65 mesh sieves, respectively. Then, approximately 5 g of corn cob or corn silk powders were transferred into 30 mL of KOH solution (6 mol L^−1^) with stirring for 12 h. The mixture obtained was washed with ultrapure water to eliminate the redundant KOH, to ensure the yield of the products. The above samples were dried at 60 °C for 24 h under vacuum, and further pyrolyzed in a tubular furnace at 700 °C for 2 h with a heating rate of 2 °C min^−1^ in a nitrogen atmosphere. Finally, the products, named as CBC and SBC, were washed with HCl (1 mol L^−1^) to remove impurities, and then washed with ultrapure water repeatedly until pH neutral, and dried at 60 °C under vacuum for 12 h.

### 2.3. Characterization

The morphology and structure of CBC and SBC were observed by field-emission scanning electron microscopy (FE-SEM) using a Zeiss Sigma 300 microscope (Oberkochen, Germany) with an XFlash 6130 instrument for the X-ray energy dispersive spectrometer (EDS). The phase and crystalline structure were characterized by X-ray diffraction (XRD) measured on a Shimadzu XRD-7000 (Kyoto, Japan) X-ray powder diffractometer with Cu Kα radiation (*λ* = 1.5406 Å). The Raman spectra were obtained from a LabRAM HR-800 Laser (HORIBA Jobin Yvon Co., Paris, France). X-ray photoelectron spectroscopy (XPS) was carried out to further analyze the elemental species and their chemical states using an ESCALAB 250Xi (Thermo Fisher Scientific, Waltham, MA, USA) apparatus. Nitrogen adsorption–desorption isotherms were performed on an ASAP 2020 V4.01 system to analyze the specific surface area and pore structure. Elemental analysis was performed on a Euro Vector EA3000 elemental analyzer (Redavalle, Italy). The S element content was determined on a sulfur determination device (Sundy SDS516, Changsha, China).

### 2.4. Electrochemical Characterization

To evaluate the electrochemical performance of the prepared materials, cyclic voltammetry (CV), galvanostatic charge-discharge (GCD) measurements and electrochemical impedance spectroscopy (EIS) were implemented on a CHI660E electrochemical workstation (Shanghai, China) in a classical three-electrode configuration. The obtained products were firstlfabricated as working electrode by mixing with acetylene black and polytetrafluorethylene (PTFE) in a mass ratio of 8:1:1, loading on a piece of pretreated nickel foam (current collector) with size of 1 cm × 1 cm under pressure of 10 MPa for 30 s, and ultimately drying at 50 °C for 12 h in a vacuum oven. The mass loading of the electrode was about 1.0–1.5 mg. In the three-electrode system, platinum foil and a saturated calomel electrode were used as the counter electrode and reference electrode, respectively, and KOH solution (2 mol L^−1^) was utilized as the electrolyte. The specific capacitance of the materials was calculated according to the GCD test results, following Formula (1):

(1) $$C=\frac{I\times \Delta t}{m\times \Delta V}$$

where *C* (F g^−1^) denoted the specific capacitance, *I* (A) referred to the charging/discharging current, Δ*t* (s) represented the discharging time, *m* (g) was the mass loading of the active material on the working electrode, and Δ*V* (V) was the applied voltage window.

For the two-electrode system, Zn foil was directly utilized as the anode, and the mixed solution of KOH (2 mol L^−1^) and Zn(Ac)_2_ (0.02 mol L^−1^) was used as the electrolyte. In order to fully study the electrochemical performance of the CBC and SBC electrodes, the area of the Zn foil in this work was larger than that of the cathode. The CV tests were performed on the CHI660E electrochemical workstation, and the GCD tests were carried out on the Land 3002A battery test system. The specific capacity (*C*, mAh g^−1^) was determined according to the following equation:

(2) $$C=\frac{I\times \Delta t}{3.6\times m}$$

where *I* (mA) was the charging/discharging current, Δ*t* (s) represented the discharging time, *m* (g) was the total mass loading of the material.

The CBC//CBC and SBC//SBC symmetric supercapacitors were assembled with identical activated porous carbon materials as the positive and negative electrodes, with KOH aqueous solution (6 mol L^−1^) as the electrolyte. The CV and GCD tests were conducted on the electrochemical workstation (CHI660E, Shanghai, China), and the long-term cycling performance was studied on the Land 3002A battery test system (Wuhan, China).

The energy density (*E*, Wh kg^−1^) and power density (*P*, W kg^−1^) of the rechargeable devices were calculated by the following equations:

(3) $$E=\frac{1}{2\times 3.6}\times C\times \Delta V^{2}$$

(4) $$P=\frac{E\times 3600}{\Delta t}$$

where *C* (F g^−1^) was the specific capacitance of the assembled devices, Δ*V* (V) was the applied voltage window, and Δ*t* (s) represented the discharging time. 

### 2.5. Computation Methods

Theoretical research studies were conducted to clarify the effects of diverse heteroatom doping on the electrochemical performance regulation of the carbon materials. All the calculations were based on density functional theory (DFT) as implemented in the Vienna ab initio simulation package (VASP) [37,38]. The ion–electron interactions were described utilizing the projector augmented wave (PAW) method [39]. The generalized gradient approximation was adopted in the Perdew–Burke–Ernzerhof (PBE) function [40,41], and the cut-off energy of the plane-wave basis was 500 eV. Prevention of the interactions between the periodic images along the *z*-direction was also considered in the DFT calculations by setting the vacuum as 15 Å. The Brillouin zone was sampled with the Monkhorst-Pack 3 × 3 × 1 and 7 × 7 × 1 k-point grids for geometry and electronic structure calculations, respectively. The energy and force convergence thresholds for the iteration in self-consistent field (SCF) were set as 10^−5^ eV and 0.02 eV Å^−1^, respectively. The van der Waals (vdW) interactions between the adsorbates and carbon materials were depicted by using Grimme’s semi-empirical DFT-D3 scheme [42]. In this study, the model of pure carbon material was constructed as the structure of graphene, where a single layer of well-ordered carbon atoms was selected. The adsorption capabilities of K* and OH* on the carbon materials were evaluated, and the adsorption energies (Δ*E*) were calculated according to the following equations:

Δ*E_K_ = E*_total_
*− E*_graphene_
*− E*_K_ (5)

Δ*E_OH_ = E*_total_
*− E*_graphene_
*−* (*E*_H_2_O_
*−* 1/2*E*_H_2__) (6)

in which *E*_total_ was the total energy of K* or OH* adsorbed on the grapheme, *E*_graphene_ was the total energy of the clean graphene without any adsorbates, and *E*_K_, *E*_H_2__, and *E*_H_2_O_ were the total energies of K, H_2_, and H_2_O free molecules in vacuum.

## 3. Results and Discussion

The elemental analysis and proximate analysis were performed to illustrate the potential of the corn cob and corn silk utilized as precursors to synthesize carbon materials. The elemental analysis results (Table S1) indicate that the corn cob and corn silk raw materials contain 42.8% and 41.1% carbon (C), respectively, which are higher than that of most other biomass [43]. Furthermore, they also contain a moderate amount of heteroatoms, which could be retained after carbonization treatment and are favorable for the improvement of the electrochemical activity of the prepared carbon materials. The corresponding results for CBC and SBC also verify that the derived carbon materials possess high carbon content and a slight amount of heteroatom doping. Meanwhile, as could be seen from the proximate analysis results in Table S2, the relatively low ash contents (2.0%) indicate the small amounts of minerals existing in the biomass, and consequently only a small quantity of derived impurities would be generated in the final calcined products. In addition, the high volatile matter contents imply large amounts of combustible substances in the raw materials, such as C, H, O, N, and S elements, which are beneficial for the uniform doping of the heteroatoms. The above results demonstrate that the selected corn wastes are suitable precursors for the synthesis of high-valued heteroatom-doped carbon materials.

**Figure 1:**
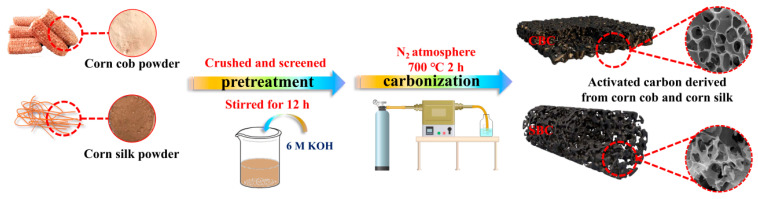
The schematic illustrations for the preparation of CBC and SBC.

The schematic illustrations for the preparation of CBC and SBC are depicted in Figure 1. The controlled synthetic processes of CBC and SBC mainly include two steps, pretreatment and carbonization. First of all, the raw corn cob and corn silk are cleaned, crushed, and screened into manageable sizes, which facilitates the permeation of the activator and the subsequent complete carbonization and activation of the materials. The gentle and long-time soaking allows the corn cob and corn silk powder to adsorb adequate KOH for activation treatment, while the consequent washing treatment with ultrapure water eliminates the redundant KOH activator and efficiently guarantees the high yield of products. Then the above samples are pyrolyzed under N_2_ atmosphere. With the release of moisture and volatile components, and the activation of KOH, a large number of micro-/meso-/macro-pores are generated. As the intrinsic properties of the biomass are different, the corn cob with relatively dense and robust structure tends to be transformed into porous carbon material with intact pore structures.

**Figure 2:**
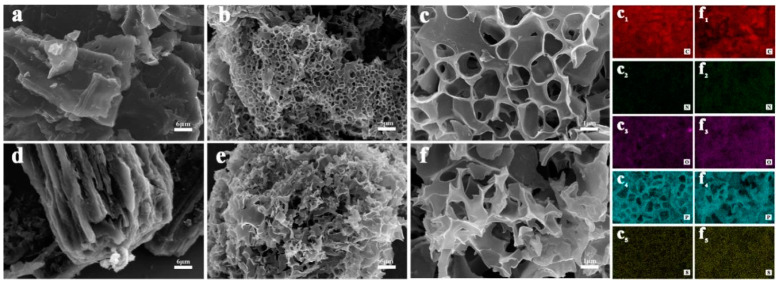
SEM images of (**a**) corn cob, (**b**) corn silk, (**c**,**d**) CBC, and (**e**,**f**) SBC. The corresponding EDS mapping results of (**c**_1_–**c**_5_) CBC and (**f**_1_–**f**_5_) SBC.

SEM characterization is performed to identify the morphology and structure of the corn cob, corn silk, CBC, and SBC. As shown in Figure 2a,d, the microstructures of the raw materials are uniform, exhibiting no impurities. Obvious hierarchical structures could be observed resulting from the transport of nutrients in the plants [44], which are suitable for the synthesis of porous biochar materials. However, compared to the corn silk with the presence of aligned channels, the corn cob mainly presents a thicker stacked texture, which could provide a more stable skeleton for harsh carbonization and activation treatments. Furthermore, the two quite different microstructures of the corn cob and corn silk make it possible to investigate the effects of the raw material properties on the pore structure regulation of the derived carbon materials and the optimization of the synthetic process. After activation and carbonization, both the CBC and SBC are rich in micropores and mesopores, as well as a certain portion of macropores, which can be seen clearly in Figure 2b,c,e,f. Comparatively speaking, the CBC possesses a porous cheese-like interconnected carbon framework with uniformly distribution of circular pores, and the SBC reveals a disorganized porous morphology with broken pore structures. Such distinctions could be attributed to the different structural characteristics of the two raw materials and the synthetic mechanism. Specifically, the formation of various pore structures is due to the fact that the KOH comes into the channels and adsorbs on the surface of the corn cob and silk during immersion, and triggers a series of redox reactions in the subsequent high-temperature carbonization process. In contrast to the relatively robust plate-like structure of corn cob, which is beneficial for the retention of intact microstructures after the harsh treatments, the formation of numerous pores results in the channel structure of corn silk becoming fragile and being more prone to collapse, eventually destroying the original regular structure. The above SEM results show that the structure features of the biomass could be utilized to adjust and control the hierarchical pore structures of the biochar.

**Figure 3:**
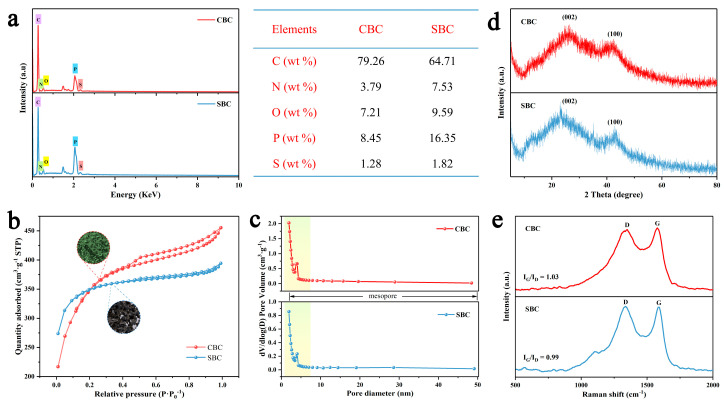
(**a**) EDS spectra, (**b**,**c**) N_2_ adsorption–desorption isotherms and BJH pore size distribution, (**d**) XRD patterns, and (**e**) Raman spectra of CBC and SBC.

The elemental composition and distribution of CBC and SBC are revealed based on the EDS results. The elemental mapping images in Figure 2(c_1_–c_5_,f_1_–f_5_) depict that the carbon (C), nitrogen (N), oxygen (O), phosphorus (P), and sulfur (S) elements are uniformly distributed in the whole framework, demonstrating the successful preparation of the multi-heteroatom doped carbon materials derived from corn wastes. The elemental composition and contents of CBC and SBC are confirmed by the EDS spectra. The spectra in Figure 3a indicate that the two carbon materials are composed of C, N, O, P, and S elements, all derived from the raw biomass materials. As shown in the corresponding Table, the element contents in CBC and SBC are quite different. The carbon content of CBC is clearly higher than that of SBC, and the nitrogen, oxygen, and phosphorus contents of CBC are lower than those of SBC. The results of the elemental analysis and the EDS exhibit similar patterns. It can be seen that even both of the precursors are obtained from corn wastes, but the element contents of the derived carbon have obvious distinctions, demonstrating that the properties of the biomass could greatly influence the composition of the derived carbon materials and their electrochemical performance as a result.

According to the SEM and EDS results, it could be reasonably deduced that the essential properties of the biomass have inevitable influences on the structure and composition design of the derived carbon materials and could dramatically affect the electrochemical performance as a consequence. Additionally, for different biomass with distinct properties, appropriate synthesis methods and conditions should be adopted and regulated. In this case, corn cob is suitable for harsh treatments with high pyrolysis temperature and strong activation of highly concentrated KOH, but corn silk with a relatively loose and unstable structure could produce porous carbon material via more gentle activation conditions. Moreover, as for the heteroatom-doping modulation of biochar materials, the intrinsic heteroatom contents of the biomass could not be neglected. Thus, the selection of suitable biomass precursors could be of great importance for the designed synthesis of the derived carbon materials.

N_2_ adsorption–desorption measurements were employed to assess the specific surface area and porous structure of the two as-prepared products and the results are shown in Figure 3b,c. Obviously, the isotherms of both CBC and SBC (Figure 3b) with the typical hysteresis loops at the *p*/*p*_0_ range of 0.4 to 0.8 are in accordance with the type IV isotherm, implying the hierarchical porous structure of the two samples [45,46]. Meanwhile, there is a sharp rise in the low relative pressure range (0–0.2), which proves the presence of a large number of micropores in the two materials. Combined with Figure 2c the pore size distribution curves show a sharp peak at about 2 nm and a narrow hump centered around 4 nm, suggesting the existence of abundant mesopores in the two structures, which is consistent with the SEM results. Thanks to the effective combination of KOH activation and high temperature carbonization treatments, the obtained CBC achieved a high specific surface area of 1149.8 m^2^ g^−1^ and a pore volume of 0.54 cm^3^ g^−1^, both of which are slightly higher than those of SBC (1083.6 m^2^ g^−1^ and 0.39 cm^3^ g^−1^). The increasing specific surface area and pore volume can accommodate more charges and shorten the ion diffusion distance during the charging–discharging process, and greatly contribute to the electrochemical performance enhancement of CBC and SBC.

XRD analysis was used to further confirm the composition and crystalline structure of the obtained materials. As shown in Figure 3d the XRD patterns of CBC and SBC possess two obvious and broad diffraction peaks at approximately 24° and 42°, corresponding to the typical (002) and (100) crystal planes of graphite carbon, respectively [47]. The peak centered at about 24° could be assigned to the formation of stacked graphite layer, and the peak sited at about 42° indicates the existence of ordered hexagonal graphite [48,49]. In addition, the XRD patterns of the two biochar materials show obvious upward warping at the 2*θ* range of 5°–10°, suggesting that both CBC and SBC possess abundant micropores, which are beneficial for the improvement of their electrochemical performance [50].

As displayed in Figure 3e the graphitic characteristics of the two samples were further examined through Raman scattering spectroscopy measurements. The two distinctive peaks in the Raman spectra correspond to the *D* and *G* bands, respectively, which are the two characteristic peaks of carbon materials. The *D* band at about 1350 cm^−1^ is related to the defective or disordered features of the graphite structure, while the *G* band at about 1590 cm^−1^ originates from the internal vibration of sp^2^-bonded carbon, relating to the well-ordered graphitic structures [51,52]. It is well known that the intensity ratio of the *G* band to *D* band (*I_G_*/*I_D_*) indicates the graphitization degree of carbon materials [53]. The values of *I_G_*/*I_D_* of CBC and SBC were calculated to be 1.03 and 0.99, respectively, indicating the presence of both ordered graphitic structures and defects in the graphitic carbon structures. For CBC, the *I_G_*/*I_D_* value is slightly higher than that of SBC, revealing a relatively higher degree of graphitization, which might increase the electrical conductivity and stability of the carbon material [53,54].

**Figure 4:**
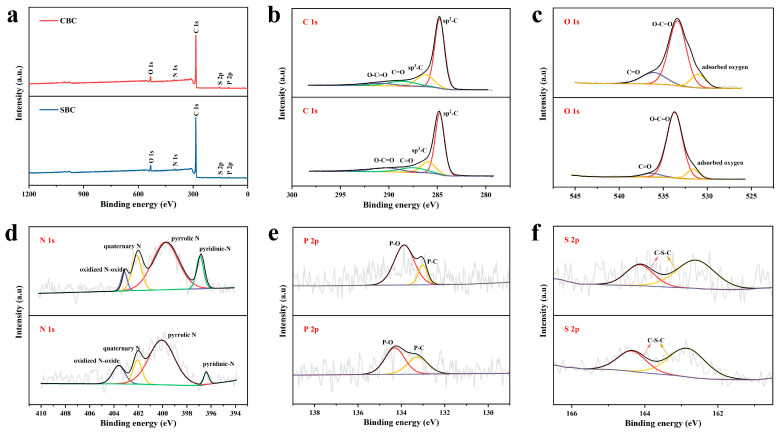
XPS spectra of CBC and SBC: (**a**) survey spectra; (**b**) C 1s; (**c**) O 1s; (**d**) N 1s; (**e**) P 2p; (**f**) S 2p.

XPS analysis was employed to reveal the surface chemical composition and different bonding states of the CBC and SBC samples. As depicted in Figure 4a, two main strong characteristic peaks namely C 1s and O 1s, and some weak peaks like N 1s, P 2p, and S 2p could be observed, which indicates the presence of O, N, P, and S heteroatoms in the as-prepared carbon materials, deriving from the original corn cob and corn silk precursors. According to previous reports, the heteroatom doping could effectively increase the defects and active sites of the carbon materials, which might be favorable for the improvement of their electrochemical activity [22,55]. The high-resolution C 1s spectra (Figure 4b) for CBC and SBC can be fitted into four individual peaks, representing the sp^2^-bonded carbon, sp^3^-bonded carbon, C=O, and O-C=O, respectively [56]. Those functional groups indicate the existence of the abundant conjugated systems, which are conducive to improving the conductivity of the porous carbon [57,58,59]. As shown in Figure 4c, the O 1s spectra of both CBC and SBC could be divided into three peaks, which are assigned to the adsorbed oxygen, O-C=O, and C=O (carbonyl oxygen of quinone), respectively [51,60,61]. The high-resolution N 1s spectra of CBC and SBC are deconvoluted into four individual peaks (Figure 4d), corresponding to the pyridinic N, pyrrolic N, quaternary N, and oxidized N-oxide species [56,62,63]. Combined with previous studies, negatively charged pyridinic N and pyrrolic N could stimulate pseudo-capacitance behavior, and positively charged quaternary N and oxidized N are believed to be beneficial to improving the electronic conductivity of materials, eventually leading to the enhancement of the electrochemical performance [63,64]. Deconvolution of the P 2p spectra reveals the presence of P-C and P-O bonds in the structure (Figure 4e) [22,56]. The high-resolution S 2p spectra of CBC and SBC can be divided into two peaks corresponding to C-S-C (Figure 4f) [22].

**Figure 5:**
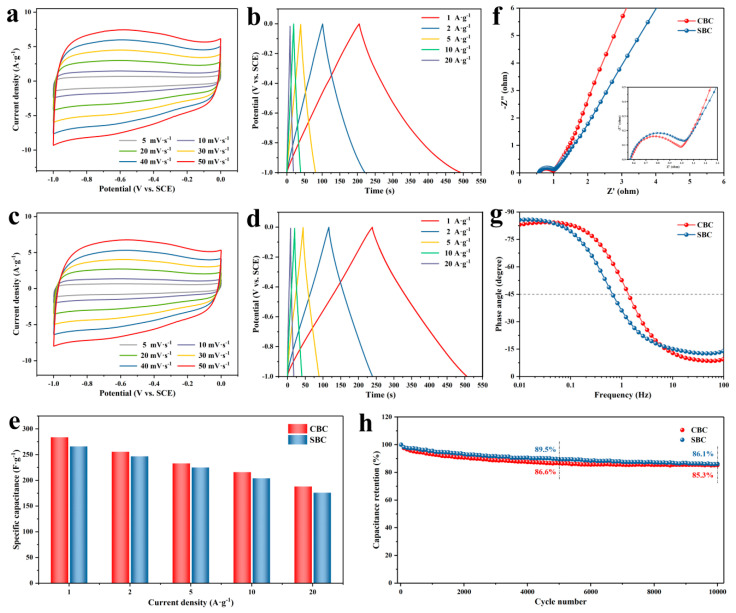
(**a**,**b**) CV and GCD curves of CBC. (**c**,**d**) CV and GCD curves of SBC. (**e**) The specific capacitances versus different current densities. (**f**,**g**) The Nyquist plots and the impedance phase angle versus frequency. (**h**) Cycling stability of CBC and SBC.

The electrochemical performance of CBC and SBC were evaluated in a classical three-electrode configuration. The CV curves of the two carbon materials at different scan rates are shown in Figure 5a,c. Obviously, all CV curves show rectangular-like shapes in the potential window of −1.0–0 V, exhibiting typical electric double layer capacitance behavior with quick electrochemical response [65]. Meanwhile, partial contribution of pseudo-capacitance could be seen from the slight bulge, which may have resulted from the doped heteroatoms. As can be seen from the curves, the slight deviation of the rectangular shape at high scanning rates is due to the response delay arising from insufficient electrolyte ion diffusion [66]. The shapes of the CV curves at different scan rates are similar without noticeable changes, revealing the excellent rate performance and high coulombic efficiency of the two samples [67]. As shown in Figure 5b,d, the GCD measurement was carried out at current densities of 1–20 A g^−1^ to further evaluate the capacitance and rate performance of CBC and SBC. The GCD curves for both CBC and SBC display typical linear and symmetrical triangular shapes, representing classical double-layer capacitance behavior, which is consistent with the CV results. Specifically, the specific capacitances of CBC were evaluated as 283.8, 255.6, 233.0, 216.0, and 188.0 F g^−1^ at the current densities of 1, 2, 5, 10, and 20 A g^−1^, respectively, while SBC exhibited specific capacitances of 266.0, 246.8, 225.0, 204.0, and 176.0 F g^−1^ at the corresponding current densities. The corresponding specific capacitances as a function of current densities are shown in Figure 5e. The results indicate that CBC and SBC have desirable specific capacitances and the great charge storage capability could still be retained at relatively high current density. Moreover, as the current density increases, the decrease of the specific capacitances is mainly due to a polarization phenomenon [53].

EIS measurement was carried out to further analyze the impedance and capacitive behavior of the electrodes. The tests were conducted at a frequency range of 0.01–100,000 Hz at open circuit potential with a current amplitude of 5 mV. According to the Nyquist impedance plots (Figure 5f), both CBC and SBC exhibit a small semicircle at high frequency and a nearly straight line at low frequency, indicating a fast charge transfer rate and an ideal capacitive behavior [68]. In the high frequency area, the value of equivalent series resistance (*R_s_*) is equal to the intercept at the *x*-axis, which involves the interface contact resistance, the inherent resistance of electrode materials, and the ionic transportation resistance of the electrolyte [69]. What is more, the diameter of the semicircle is related to the charge transfer resistance (*R_ct_*), and the slope of the slanted line at low frequency is relevant to the Warburg impedance (*W_z_*), arising from the dispersion of electrolyte ions in the active materials [70,71]. It can be clearly seen from the image that the *R_s_* values of CBC and SBC are 0.58 and 0.57 Ω, indicating that both electrodes possess low intrinsic resistance and interface contact resistance. In addition, the plot for CBC has a relatively smaller semicircle diameter and larger slope, demonstrating the lower transportation resistance of electron and electrolyte ion during the charge storage process. Figure 5g shows the Bode plots of impedance phase angle versus frequency for the CBC and SBC. The phase angle of nearly 90° could be observed from the plots at a frequency of 0.01 Hz, revealing the ideal capacitive behavior of the two samples. Meanwhile, the values of characteristic frequency (*f*_0_) of CBC and SBC at −45° are 1.35 and 0.68 Hz, corresponding to the relaxation time constant *τ*_0_ of 0.74 and 1.47 s, respectively, which represents the faster charge migration of CBC compared to SBC [56].

The cycling performances of CBC and SBC were also evaluated by calculating the initial specific capacitance retention after 10,000 cycles. As shown in Figure 5h, the specific capacitances of CBC and SBC could maintain 85.3% and 86.1%, respectively, highlighting the superior charge–discharge stability of the two porous carbon electrodes. Considering the electrical double-layer capacitance behavior of CBC and SBC, the excellent stability of the two carbon materials could be attributed to their high specific surface area and desired pore size distribution, which could accommodate the migration and adsorption of electrolyte ions. All the above electrochemical results reveal that the structure and composition strengths of the carbon materials prepared in this work greatly regulated their electrochemical performance.

**Figure 6:**
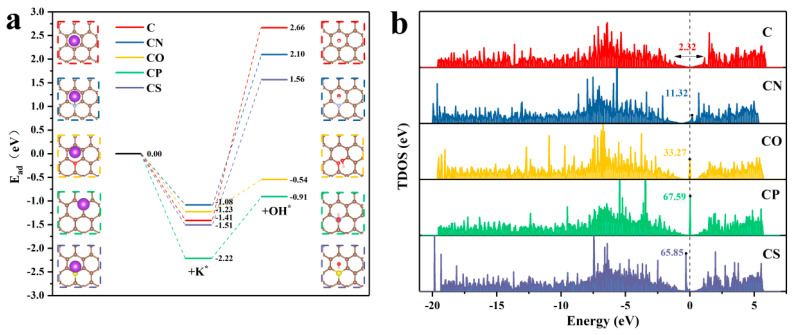
(**a**) Adsorption ability of K* and OH* on pure carbon and heteroatom doped carbon materials. The brown, gray, red, light pink, yellow, purple, and light red balls represent C, N, O, P, S, K, and H atoms, respectively. (**b**) TDOS diagrams of the pure carbon and the heteroatom doped carbon materials.

In order to further elucidate the enhanced electrochemical properties of CBC and SBC, the effects of different heteroatom doping on their electrochemical performance were investigated by adopting density functional theory (DFT) calculations. Based on the EDS and XPS characterizations, the N, O, P, and S doped carbon models were constructed individually, and for comparison, a pure carbon model was also designed. The top view optimized models are shown in Figure S1. Considering the double electric layer charge storage mechanism, the carbon-based materials store energy is mainly based on the adsorption/desorption of electrolyte ions and since the adsorption energies of K* and OH* are relevant to the reaction kinetics during the charge–discharge process [55], the adsorption energy of the electrolyte ions (K* and OH*) on the carbon materials with different heteroatom doping was calculated. The optimized models for the adsorption of K* and OH* and the specific adsorption free energies are shown in Figure 6a. As can be seen, the adsorption energies of K* on the P or S doped carbon materials are lower than that on the pure carbon, especially for the P doped carbon, which has the most superior K* adsorption ability. The lower adsorption energies indicate that the materials have a higher affinity for K* [72]. Meanwhile, the adsorption energies of OH* on the materials doped with N, O, P, or S atoms are 2.10, −0.54, −0.91, and 1.56 eV, respectively, which are lower than that on the pure carbon material (2.66 eV). The electrical conductivity of the pure carbon and the heteroatom doped carbon materials was also evaluated, and the results are shown in Figure 6b. It is obvious that the doping of heteroatoms could greatly optimize the conductivity of the materials, and the P and S doping could lead to a noticeably more positive effect. These results reaffirm that heteroatom doping contributes to additional active sites on the materials and could effectively improve the electrical conductivity and the affinity of the prepared materials for electrolyte ions, which have been proven to be more favorable for the improvement of the electrochemical properties [73]. In addition, the biochar materials synthesized in this work were multi-heteroatom doped, which might have had a synergistic promotion effect on their electrochemical performance regulation.

**Figure 7:**
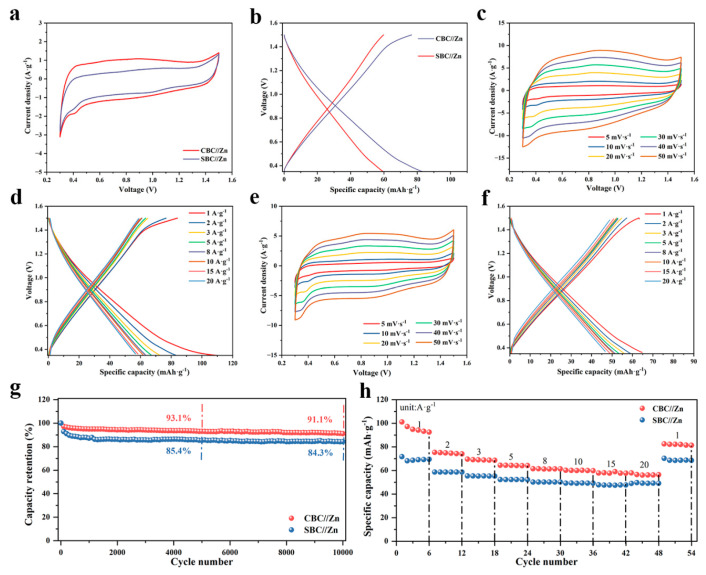
(**a**,**b**) The CV and GCD comparisons of the CBC//Zn and the SBC//Zn. (**c**,**d**) The CV and GCD curves of the CBC//Zn. (**e**,**f**) The CV and GCD curves of the SBC//Zn. (**g**,**h**) The cycling performance and rate performance of the CBC//Zn and the SBC//Zn.

Zinc batteries generally suffer from poor rate performance and cycling stability. In this work, we utilized creatively biochar-based cathodes to solve the above problems. The electrochemical performance of the CBC and SBC cathodes was evaluated in a two-electrode system using a Zn anode and an alkaline-based electrolyte. As shown in Figure 7a,b, the CV curve (at 5 mV s^−1^) of the CBC//Zn has a slightly larger integrated area compared to that of the SBC//Zn, and the GCD curve (at 2 A g^−1^) of the CBC//Zn also exhibits the longer discharging time, both of which indicate that the CBC cathode presents higher specific capacity. As can be seen in Figure 7c,e, the CV curves of the CBC//Zn and the SBC//Zn are mainly rectangular shapes, which implies the primary capacitive contribution during the charge storage process. In Figure 7d,f, the CBC//Zn delivers high specific capacities of 109.6, 83.2, 72.8, 67.2, 63.1, 61.7, 58.3, and 56.7 mAh g^−1^ at 1, 2, 3, 5, 8, 10, 15, and 20 A g^−1^, respectively while the SBC//Zn shows specific capacities of 67.9, 59.6, 56.5, 53.9, 51.6, 50.6, 49.2, and 46.7 mAh g^−1^ at the same current densities. The above results reveal that the CBC has relatively higher specific capacities, and both of the two cathodes show desirable rate performance. In Figure 7g, it is remarkable that both of the two zinc batteries present superior cycling stability with 91.1% and 84.3% specific capacity retention after 10,000 cycles, respectively, which highly demonstrates the great merits of using the CBC and SBC biochar-based materials as the zinc battery electrodes. The rate performance of the two cathodes was further studied and the results are shown in Figure 7h. The CBC delivers a higher specific capacity at 1 A g^−1^, but relatively lower capacity retention at 20 A g^−1^ compared with that of the SBC. However, the capacities of the CBC undergo slight decreases from 2 A g^−1^ to 20 A g^−1^, which also manifests the great rate performance of the cathode. For the SBC cathode, the specific capacity could be retained about 68.3% at a high current density of 20 A g^−1^, and the capacity also became stable from 2 A g^−1^ to higher current densities. Moreover, as the current density was set back to 1 A g^−1^, the specific capacities of CBC//Zn and SBC//Zn could be almost maintained at the same level compared with those in the initial stage. The excellent cycling stability and rate performance of the CBC and SBC cathodes could be attributed to the primary capacitive contribution during the charging–discharging process. Biochar-based materials have been rarely reported as being used as zinc battery cathodes, but in this work, the as-prepared CBC and SBC showed excellent charge storage capability, which also provides a new perspective in the development of high-performance rechargeable devices.

**Figure 8:**
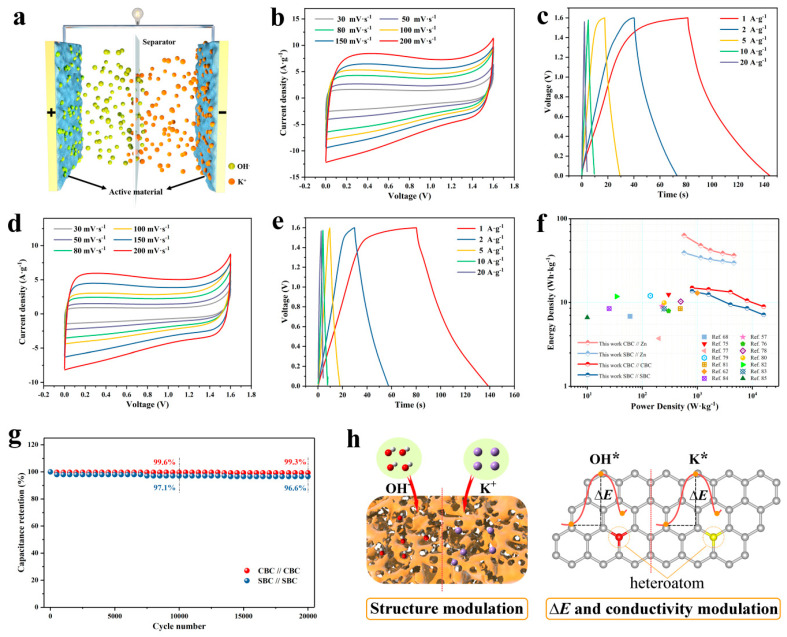
(**a**) Schematic illustration of the symmetric supercapacitors. (**b**,**c**) CV and GCD curves of CBC//CBC. (**d**,**e**) CV and GCD curves of SBC//SBC. (**f**) Ragone plots of the symmetric supercapacitors. (**g**) Cycling performance of the symmetric supercapacitors. (**h**) Schematic illustration of the charge storage process of CBC and SCB.

In order to further assess the electrochemical performance and practical application of the prepared materials, CBC and SBC were employed as electrodes to fabricate symmetric supercapacitors (CBC//CBC and SBC//SBC), and the schematic illustration of the symmetric supercapacitors is shown in Figure 8a. According to the equation, *E* = 1/2*CV*^2^, it is evident that high potential value is of great significance for the improvement of the energy density of the supercapacitors [74]. The CV curves of the above devices performing under different voltage windows (0.8–1.8 V) are shown in Figure S2. To minimize the effects of polarization during the tests, the operating voltage of the supercapacitors was finally set as 1.6 V. Figure 8b,d displays the CV curves of the two supercapacitors at scan rates ranging from 30 to 200 mV s^−1^. Obviously, all the CV curves reveal ideal capacitive behavior with symmetrical rectangular shapes. It is noteworthy that satisfactory rectangular curves can still be observed at the high scan rate of 200 mV s^−1^, implying the rapid charge transfer capability and great rate performance of the assembled supercapacitors. Furthermore, Figure 8c,e displays the GCD curves of the assembled supercapacitors at diverse current densities of 1, 2, 5, 10, and 20 A g^−1^, and the corresponding specific capacitances of CBC//CBC are 41.9, 40.2, 37.5, 29.4, and 25.1 F g^−1^, respectively. While the SBC//SBC possesses specific capacitances of 38.1, 35, 26.3, 23.8, and 20 F g^−1^.

Figure 8f summarizes the energy densities and power densities of CBC//Zn, SBC//Zn, CBC//CBC, SBC//SBC, and some previously reported devices in the form of Ragone plots. It is evident that the zinc batteries show much higher energy densities than those of the supercapacitors. The energy densities of the CBC//Zn and the SBC//Zn are 63.0 and 39.1 Wh kg^−1^ at a power density of 575 W kg^−1^, respectively. At a much higher power density of 4600 W kg^−1^, their energy densities could still be retained at 36.3 and 29.7 Wh kg^−1^. As for the supercapacitors, the CBC//CBC achieves a high energy density of 14.9 Wh kg^−1^ at a power density of 800 W kg^−1^, and the SBC//SBC delivers an energy density of 13.6 Wh kg^−1^. Even at the high-power density of 16,000 W kg^−1^, the CBC//CBC and SBC//SBC can maintain energy density of 8.9 Wh kg^−1^ and 7.1 Wh kg^−1^, respectively. It is noticeable that the energy densities of the as-assembled rechargeable devices in this work are higher than the commercial carbon-based supercapacitors (4–6 Wh kg^−1^) and most of the other reported biochar-based devices [56,62,68,75,76,77,78,79,80,81,82,83,84,85]. The cycling stability of the symmetric supercapacitors was also assessed and the results are shown in Figure 8g. Remarkably, the specific capacitances of CBC//CBC and SBC//SBC still maintain 99.3% and 96.6% after cycling for 20,000 times with almost no capacitance decay, suggesting the outstanding long-term cycling stability, superb reversibility, and high practical application potential of the assembled supercapacitors. In summary, the above electrochemical test results fully verified the superior electrochemical performance of the obtained electrodes and their corresponding fabricated rechargeable devices. As shown in Figure 8h the excellent electrochemical performance of CBC and SBC could be mainly attributed to their hierarchical porous structure and the multi-heteroatom doped composition features. The hierarchical porous structure could highly facilitate the electrolyte ion transportation and expose more accessible surface area for the electrostatic adsorption of charged ions. Additionally, the doped heteroatoms inherited from the raw corn wastes could dramatically optimize the adsorption energies for the K* and OH* and the electrical conductivity of the prepared biochar materials, which could provide more active sites than the pure carbon materials and efficiently enhance their electrochemical activity as a consequence.

## 4. Conclusions

In this work, corn wastes were adopted to synthesize controllably high-performance porous and multi-heteroatom doped carbon materials in high yield. These were also utilized as high-performance cathodes in rechargeable devices, especially being used creatively in zinc batteries. The experimental and theoretical investigation results revealed that the essential properties of the biomass had negligible influences on the composition and nanostructure regulation of the derived carbon materials and their electrochemical performance. Additionally, the multi-heteroatoms doping of the carbon materials could efficiently optimize the adsorption energies of K* and OH*, and noticeably increase the electrical conductivity, which proved to be of great significance for their electrochemical performance improvement. The assembled CBC//Zn and SBC//Zn zinc batteries deliver high energy densities of 63.0 Wh kg^−1^ and 39.1 Wh kg^−1^ at a power density of 575 W kg^−1^, and excellent cycling performance of 91.0% and 84.3% capacitance retention after 10,000 cycles. The symmetric supercapacitors also possess relatively desirable energy and power densities, as well as superior long-term cycling stability. This study has provided a facile and economical way to produce high value-added corn waste derived electrode materials with excellent electrochemical performance. Furthermore, the investigation method laying emphasis on the effects of biomass properties on the electrode performance modulation has a guiding significance for the design and application of other biochar electrodes.

## Data Availability

The data that support the findings of this study are available from the corresponding author, upon reasonable request.

## Supplementary Materials

The following supporting information can be downloaded at: https://www.mdpi.com/article/10.3390/ma16145022/s1, Figure S1: The optimized top view models of the pure carbon and the heteroatom doped carbon materials, in which the brown, gray, red, light pink and yellow balls represent C, N, O, P and S atoms, respectively. Figure S2: The CV plots of (a) CBC//CBC and (b) SBC//SBC under different potential windows at a scan rate of 80 mV s^−1^; Table S1. Elemental analysis for CBC, SBC, corn cob and corn silk. Table S2: Proximate analysis for the corn cob and corn silk.
