# Supplementary material for: Electrochemical Performance of Corn Waste Derived Carbon Electrodes Based on the Intrinsic Biomass Properties
Source: Materials (Basel). 2023 Jul 15;16(14):5022. doi: 10.3390/ma16145022 (PMC10384028; doi:10.3390/ma16145022)
Supplement: Supplementary file 1 [file materials-16-05022-s001.zip › materials-2422402-supplementary.docx]

Supporting Information

**Electrochemical Performance of Corn Waste Derived Carbon Electrodes Based on the Intrinsic Biomass Properties**

Kunhan Xie^1,†^, Wen Zhang^1,†^, Kai Ren^1^, Enze Zhu^1^, Jianyi Lu^1^, Jingyang Chen^1^, Penggang Yin^2^, Liu Yang^1,^*, Xiaohui Guan^1,^*, Guangsheng Wang^2^

^1^ Jilin Provincial Science and Technology Innovation Center of Clean Conversion and High-Valued Utilization of Biomass, School of Chemical Engineering, Northeast Electric Power University, Jilin 132012, P. R. China

^2^ School of Chemistry, Beihang University, Beijing 100191, P. R. China

^†^ These authors contributed equally to this work.

^*^ Corresponding Authors: [l.yang@neepu.edu.cn](mailto:l.yang@neepu.edu.cn), [guanxh@neepu.edu.cn](mailto:guanxh@neepu.edu.cn)


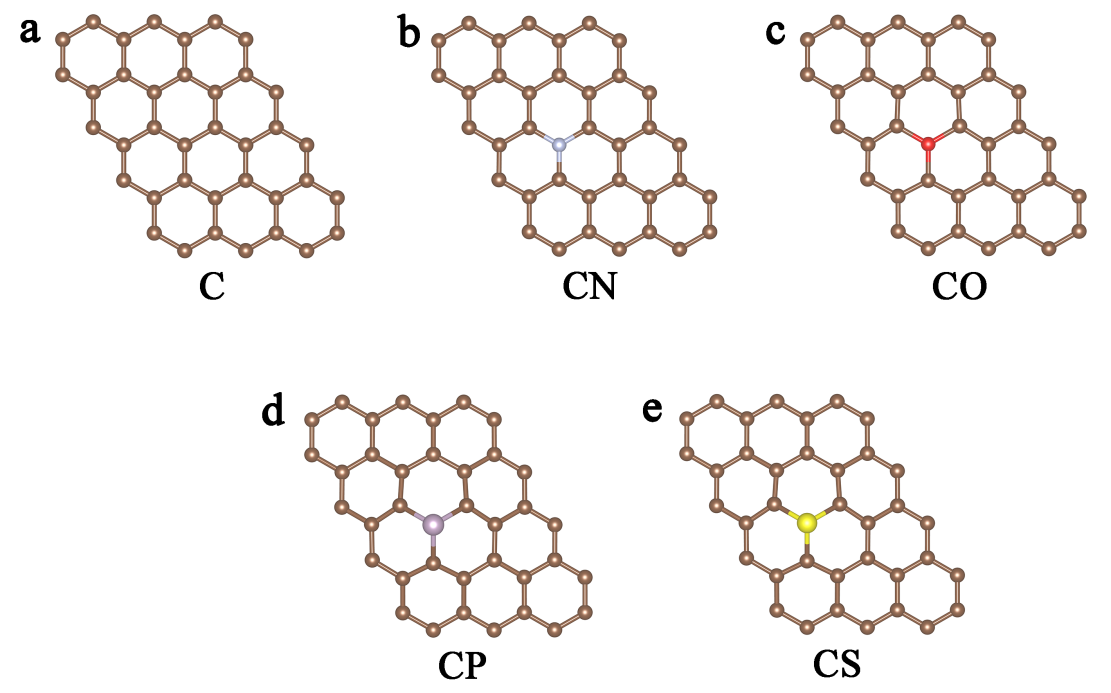


**Figure S1.** The optimized top view models of the pure carbon and the heteroatom doped carbon materials, in which the brown, gray, red, light pink and yellow balls represent C, N, O, P and S atoms, respectively.


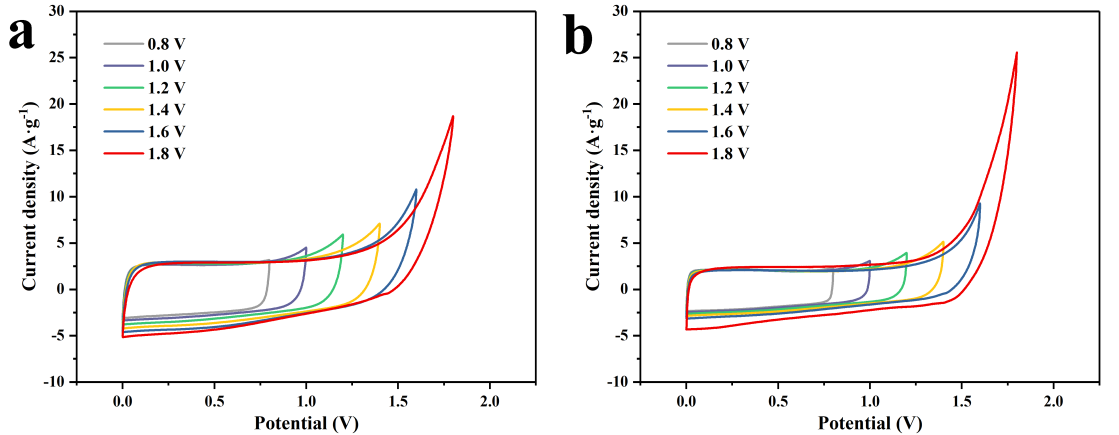


**Figure S2.** The CV plots of (a) CBC//CBC and (b) SBC//SBC under different potential windows at a scan rate of 80 mV s^-1^.

**Table S1.** Elemental analysis for CBC, SBC, corn cob and corn silk.

| Samples | C (%) | H (%) | N (%) | S (%) |
| --- | --- | --- | --- | --- |
| CBC | 77.4 | 2.8 | 0.8 | 0.3 |
| SBC | 73.4 | 2.7 | 3.1 | 0.4 |
| Corn cob | 42.8 | 5.9 | 3.6 | 0.5 |
| Corn silk | 41.1 | 6.6 | 3.6 | 0.5 |

**Table S2.** Proximate analysis for the corn cob and corn silk.

| Samples | M_ad_ (%) | A_ad_ (%) | A_d_ (%) | V_ad_ (%) | V_d_ (%) | V_daf_ (%) | FC_ad_ (%) | FC_d_ (%) |
| --- | --- | --- | --- | --- | --- | --- | --- | --- |
| Corn cob | 7.4 | 2.0 | 2.0 | 73.7 | 79.6 | 81.2 | 17.0 | 18.4 |
| Corn silk | 8.3 | 2.0 | 2.2 | 73.0 | 79.5 | 81.3 | 16.8 | 18.3 |
